# Effect of legumes in energy reduced dietary approaches to stop hypertension (DASH) diet on blood pressure among overweight and obese type 2 diabetic patients: a randomized controlled trial

**DOI:** 10.1186/s13098-022-00841-w

**Published:** 2022-05-13

**Authors:** Somayeh Hosseinpour-Niazi, Farzad Hadaegh, Parvin Mirmiran, Maryam S. Daneshpour, Maryam Mahdavi, Fereidoun Azizi

**Affiliations:** 1 Nutrition and Endocrine Research Center, Research Institute for Endocrine Sciences, Shahid Beheshti University of Medical Sciences, Tehran, Iran; 2 Prevention of Metabolic Disorders Research Center, Research Institute for Endocrine Sciences, Shahid Beheshti University of Medical Sciences, Tehran, Iran; 3 Cellular and Molecular Research Center, Research Institute for Endocrine Sciences, Shahid Beheshti University of Medical Sciences, Tehran, Iran; 4 Obesity Research Center, Research Institute for Endocrine Sciences, Shahid Beheshti University of Medical Sciences, Tehran, Iran; 5 No. 24, A’rabi St., Yeman Av., Velenjak, P.O. Box: 19395-4763, Tehran, Iran

**Keywords:** Legumes, DASH diet, Blood pressure, Type 2 diabetes

## Abstract

**Background:**

This study aimed to investigate the effects of legumes in dietary approaches to stop hypertension (DASH) on blood pressure and urinary sodium and potassium in participants with type 2 diabetes. We further investigated whether changes in individual dietary food groups in the DASH diet contribute to blood pressure.

**Methods:**

Participants were randomized to the traditional DASH diet (n = 150) or the legume-based DASH diet (n = 150). Blood pressure and urinary sodium and potassium were measured at baseline and after 16-week interventions. An intention-to-treat approach with multiple imputations of missing data was applied. The restricted cubic spline (RCS) was applied to assess the linearity and explore the shape of the relationship between the changes in food groups and systolic blood pressure (SBP) in the two intervention groups.

**Results:**

A reduction in SBP and urinary sodium was observed at week 16 in both dietary interventions, and this reduction was more significant in the legume-based DASH diet, than in the DASH diet. In the legume-based DASH diet, SBP decreased with the legume intake of more than 95.8 g/day (*P* nonlinear < 0.001). The multivariable RCS analysis showed a U-shaped relationship of refined grains, an L-shaped relationship of fruits, and a linear relationship of sweet, energy, sodium and vegetables intake with SBP.

**Conclusions:**

The DASH diet, enriched in legumes, could improve SBP in participants with type 2 diabetes. In DASH diet, the balance of consumption of refined grains and fruits along with higher vegetable consumption and lower sweet, sodium and energy intake reduced the SBP.

**Trial registration:** IRCT20090203001640N17.

## Introduction

Hypertension is a public health crisis, affecting more than one in four adults globally [1]. It is more common in type 2 diabetic patients with a prevalence of 70% [2]. Compared with non-diabetic individuals, patients with type 2 diabetes have nearly twice the incidence of hypertension [3]. The overlap between type 2 diabetes and hypertension markedly increased cardiovascular disease (CVD)-related morbidity and mortality [4]. The UK Prospective Diabetes Study showed that among type 2 diabetes patients, a 10 mg reduction in systolic blood pressure reduced the risk of CVD morbidity and mortality by 6–12% in type 2 diabetes patients [5].

Dietary modification can be effective in controlling and preventing diabetes and hypertension [6–9]. The Dietary Approaches to Stop Hypertension (DASH) diet, which is rich in healthy food groups, was developed to prevent and treatment of hypertension [10]. Also, finding from the optimal macronutrients intake (OMNI)-Heart trial which modified the DASH diet by replacing 10% of the carbohydrate with vegetable-based protein indicated a further reduction in blood pressure, compared with the original DASH diet among hypertensive patients [11]. However, replacing other animal sources of protein with red meat protein in DASH diet resulted in no more improvement in blood pressure among healthy [12], and hypertensive subjects [13].

Dietary guidelines have emphasized reducing the intake of red and processed meat and replacing these food groups with high-quality plant-based protein sources, such as legumes [14, 15]. However, it is unclear whether replacing red meat with legumes is associated with a reduced risk of hypertension [16, 17]. In addition, the majority of previous studies were conducted among healthy individuals or participants with hyperlipidemia or hypertension, and few studies investigated the effect of legumes on blood pressure among participants with type 2 diabetes; these studies showed that an 8-week consumption of legumes, 4–6 servings per week, did not have a significant effect on blood pressure among type 2 diabetes patients or individuals at risk of type 2 diabetes [18–20]. The low consumption rate of legumes compared to the recommended amount [17], as well as the small sample size, and the short follow-up period prevented the possible beneficial effect of legumes on blood pressure in these populations [18–20]. Therefore, this randomized controlled trial (RCT) aimed to investigate the effects of replacing one serving of red meat with legumes for at least 5 days per week in the DASH diet on blood pressure over 16 weeks in overweight and obese participants with type 2 diabetes. We further investigated which dietary food groups in the DASH diet were mostly contributed to the blood pressure reduction.

## Methods

This RCT was registered in the Iranian Registry of Clinical Trials (IRCT) (code: IRCT20090203001640N17). The Ethics Committee of the Research Institute for Endocrine Sciences, affiliated with Shahid Beheshti University of Medical Sciences, Tehran, Iran, approved the study design (No.: IR.SBMU.ENDOCRINE.REC.1399.001) before participant recruitment. Also, all participants provided written informed consent prior to recruitment.

### Design and setting of the study

This RCT was performed in the framework of the Tehran Lipid and Glucose Study (TLGS). From July 11, 2020, to March 10, 2021, in the TLGS unit, we recruited type 2 diabetes subjects (FPG level ≥ 126 mg/dl or the two-hour plasma glucose level ≥ 200 mg/dL or oral hypoglycemic medication consumption) aged 30–65 years, with BMI of 25–40 kg/m^2^, no weight change from three months before enrollment in the study, red meat consumption ≥ 1 serving/day, and willingness to consume legumes in the diet. Participants were excluded if they were pregnant or lactating, had a renal failure (creatinine ≥ 1.4 mg/dL in men and ≥ 1.3 mg/dL in women), or used insulin. According to the Declaration of Helsinki principles, the study procedure, objectives, and adverse events were explained to each participant.

### Randomization and allocation concealment

An independent staff member of the TLGS randomly assigned the participants to the DASH diet or the legume-based DASH diet using block randomization sequences (four participants per block) generated by the randomization website (www.randomization.com).

Allocation to intervention was concealed from the principal investigators using sequentially numbered sealed opaque envelopes, which were only opened sequentially by a dietician in the presence of eligible participants at the first visit.

### Dietary interventions

All participants were advised to complete a run-in period for 2 weeks, including adherence to their diet while eliminating legumes. Participants not willing to continue the study were excluded. Eligible participants were randomly allocated to the DASH diet or legume-based DASH diet over 16 weeks. The energy requirements of each participant were estimated from the initial resting energy expenditure, based on the Mifflin-St Jeor equation, multiplied by the physical activity coefficient.

Because the participants were overweight and obese, dietary interventions were designed to provide 500–700 kcal/day less than their energy requirement. Both dietary interventions provided almost 25–30% of the energy from fat, 15% from proteins, and 55–60% from carbohydrates. In the DASH diet group, the participants were instructed to follow the DASH diet (2000–3000 kcal based on energy requirement), which consisted of 8–12 servings/day of fruits and vegetables, 7–15 servings of whole grains, 2–3 servings of low-fat dairy products, two servings of red meat, one serving of nuts and seeds, and limited intake of sweets and sugar (five servings per week).

The legume-based DASH diet was similar to the traditional DASH diet, but one serving of red meat was replaced with one serving of legumes at least 5 days per week. Also, because legumes are equivalent to one serving of whole grains, one serving of bread was eliminated from the legume-based DASH diet. In both diets, the participants were advised to consume one teaspoon of salt per day (2300 mg/d) or less. All participants were asked to maintain their physical activity and not change their medications during the 16-week interventions unless prescribed by their physician. During the study, no changes in medication use were found in any participants.

Every 2 week, the dietitians called the participants to gather their dietary information using 3 day food records (2 weekdays and 1 weekend day) during the run-in period and the study period. Each phone call took between 45 and 60 min. Of 300 participants, 269 (89.6) completed 40–48 food records, and 31 (10.6) completed 30–39 food records during 16-week interventions.

Dietary intake include the mean intake of food records during 16 weeks follow-up. Dietary intake of fruits and vegetables, whole grains, refined grains, low-fat dairy products, red meat, nuts and seeds, sweets, legumes, salt intake, and energy intake were determined. To evaluate the adherence to dietary interventions, the dietitian compared participants' dietary intake with the dietary instructions, and reinforced their dietary adherence. The intake of macro- and micronutrients was also calculated using NUTRITIONIST III version 7.0 (N-Squared Computing, Salem, OR, USA), designed for Iranian food.

### Measurements

Weight was measured with minimal clothing without shoes using a digital scale (Seca 707; range 0–150 kg) with an accuracy of 100 g. After a 15 min rest, blood pressure was measured using a standardized mercury sphygmomanometer on the right arm twice, at least 30 s apart. The average of the two measurements was reported as the subject’s blood pressure. Spot urine samples were obtained between 7:00 and 9:00 a.m. following overnight fasting. Urinary concentrations of sodium (mmol/dL) and potassium (mmol/dL) were measured using flame photometry (Screen Lyte, Hospitex Diagnostics, Florence, Italy).

### Definition of terms

Hypertension was defined as systolic blood pressure (SBP) ≥ 140 mmHg and/or a diastolic blood pressure (DBP) of ≥ 90 mmHg or treatment with anti-hypertensive drugs [21]. Hyperlipidemia was defined as LDL-C ≥ 150 mg/dL, TC ≥ 200 mg/dL, and/or HDL-C < 40 mg/dL, and/or a triglyceride ≥ 150 mg/dL, and/or treatment with lipid lowering drugs [22].

### DASH diet score calculation

We calculated a DASH diet score, based on eight dietary components, including fruits and vegetables, legumes and nuts, whole grains, low-fat dairy products, red and processed meat, sweets, fish and sodium [23].

### Statistical analysis

With a two-sided α error = 0.05 and β error = 0.20 and 10 mmHg (SD = 16.6) reduction in SBP (minimal clinically important difference), and by assuming an attrition rate of 20%, total sample size was estimated to be 300 participants [20, 24] using following formula: ((Z _1-α/2_ + Z_1-β_)^2^ * (S_1_^2^ + S_2_^2^))/$\Delta$ ^2^.

All analyses were performed using Stata version 14.0 software (StataCorp LLC, TX, USA). Analyses were performed on all participants who were randomly allocated to the dietary interventions based on the intention-to-treat (ITT) principle. The Multiple imputation by chained equations (MICE) procedure was applied to impute the missing data (n = 16) for the outcomes. In multiple imputations, the predictors included all variables in Table 1, as well as dietary interventions. The baseline characteristics are presented as mean ± SD for demographic variables, dietary factors, SBP, DBP, and urine electrolytes and count (percentage) for dichotomous variables. Comparisons of food groups between interventions diets were performed using analysis of covariance (ANCOVA) adjusted for energy intake. The ANCOVA that adjusted for baseline values, oral antidiabetic medications, antihypertensive medications, and changes in physical activity levels, was also used to assess if the changes (week 16–baseline) in SBP, DBP, urinary sodium, urinary potassium, and the urinary sodium to potassium ratio were significantly different between the dietary*.* All statistical tests were considered statistically significant when *P* value was < 0.05. The restricted cubic spline (RCS) was applied to assess the linearity and explore the shape of the relationship between dietary food groups and SBP in two intervention groups.

**Table 1:** Baseline characteristic of participants according to group of intervention diets

Characteristics	Total population	
	DASH diet	Legume-based DASH diet
Age, years	55.3 (6.9)	55.4 (7.1)
Female, n (%)	84 (56.0)	87 (58.0)
BMI, kg/m^2^	30.7 (3.4)	30.7 (3.6)
Physical activity levels, Met-h/week	3.5 (2.7)	3.4 (2.7)
Academic degree, n (%)	18 (12.0)	22 (14.7)
Hypertensive participants^*^, n (%)	90 (60.0)	85 (56.7)
Hyperlipidemia participants^**^, n (%)	135 (90.0)	140 (93.3)
Medication		
Oral antidiabetic medications		
Metformin, n (%)	55 (36.7)	69 (46.0)
Sulfonylurea, n (%)	40 (26.7)	29 (19.3)
Metformin + sulfonylurea, n (%)	45 (30.0)	31 (20.7)
Metformin + thiazolidinedione, n (%)	5 (3.3)	11 (7.3)
Others, n (%)	5 (3.3)	10 (6.7)
Antihypertensive drugs		
ACE inhibitor/ARB use, n (%)	52 (34.7)	48 (32.0)
Thiazide, n (%)	7 (4.7)	2 (1.3)
Others, n (%)	10 (6.7)	8 (5.3)
Lipid lowering drugs		
Statin use, n (%)	85 (56.7)	85 (56.7)
Others, n (%)	1 (0.7)	4 (2.7)

*DASH* dietary approaches to stop hypertension, *BMI* body mass index, *Met-h/week* metabolic equivalent (MET)-hours per week

Data are mean (SD) unless otherwise indicated

^*^BP ≥ 140/90 or treatment with anti-hypertensive medications

^**^ LDL-C ≥ 150 mg/dL, TC ≥ 200 mg/dL, and/or HDL-C < 40 mg/dL, and/or a triglyceride ≥ 150 mg/dL, and/or treatment with lipid lowering drugs

## Results

**Fig. 1:**
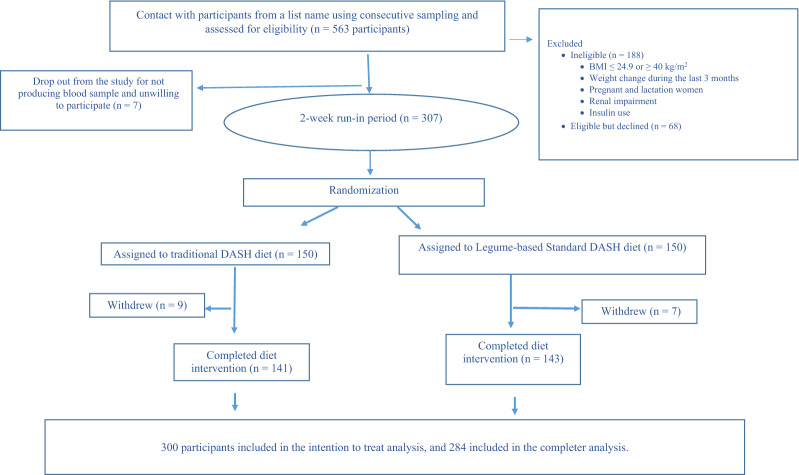
The flow diagram of the study

Between July 11, 2020, and March 10, 2021, a total of 563 participants were screened, of whom 307 met the inclusion criteria, and 300 were randomized to the dietary interventions (Fig. 1). Finally, 284 completed the study. The mean (SD) age and BMI were 55.4 (7.0) years and 30.4 (3.4) kg/m^2^, respectively. Besides, 57.1% were male, and 48.7% were obese. At baseline, the median DASH diet score was 3.0 (interquartile range 2–4) in both intervention groups. The baseline characteristics were similar between the dietary intervention groups, except for oral antidiabetic medication; in the legume-based DASH diet, the percentage of participants treated with metformin was slightly higher than in the DASH diet group (Table 1). Physical activity did not change during the study in both groups [3.5 (2.7) Met-h/week at baseline vs. 3.4 (2.7) at end of trial in DASH diet; 3.4 (2.7) at baseline vs. 3.4 (2.7) at end of trial in legume-based DASH diet].

No significant differences were observed between the intervention groups for dietary intake at baseline. Analysis of the subjects’ food records during dietary interventions showed a significant reduction in the red meat consumption (mean change difference between diets −41.0, 95% CI −63.7 to −18.5 g/day, P < 0.001), saturated fatty acid (−1.6, −1.9 to −1.2, *P* < 0.001), and cholesterol (−20.9, −37.3 to −4.4, *P* = 0.012) in the legume-based DASH diet compared to the DASH diet. Legume consumption (100.4, 96.7–104.1, *P* < 0.001) and fiber intake (5.1, 2.0–8.1, *P* = 0.001) increased significantly in the legume-based DASH diet compared to the DASH diet. No significant differences in fruit, vegetables, whole grains, refined grains, nuts and seeds, low-fat dairy products, sweets, sodium intake, DASH score, carbohydrate, protein, fat and sodium intake were found between the two groups (Table 2). Weight decreased after consumption of both hypocaloric dietary interventions [−4.0 (−5.0 to −3.0) in DASH diet and −3.7 (−7.4 to −2.7] in legume-based DASH diet); however, the difference between two dietary interventions was not significant (Table 2).

**Table 2:** Baseline and the 16-week change in dietary intake after the DASH diet and legume-based DASH diet, based on intention to treat analysis

	DASH diet (n = 150)		Legume-based DASH diet (n = 150)		Difference in change	
	Run-in-period	Change from baseline	Run-in-period	Change from baseline	Mean (95% CI)	*p *value
DASH score	3.1 (1.2)	2.7 (2.3)	3.1 (1.4)	3.1 (2.3)	0.4 (−0.12–0.92)	0.133
Red meat (g/day)	57.0 (52.3)	6.8 (98)	64.9 (72.8)	−34.3 (101)	−41 (−63.7–−18.5)	*P* < 0.001
Legumes (g/day)	0 (0)	13.6 (15.0)	0 (0)	114 (17.2)	100.4 (96.7–104.1)	*P* < 0.001
Fruit (g/day)	268 (183)	120 (167)	296 (191)	120 (152)	0.0 (−3.6.2–36.2)	1.0
Vegetables (g/day)	141.1 (102)	63.9 (90.5)	131.3 (99.1)	67.6 (81.0)	3.7 (−15.8–23.2)	0.070
Whole grains (g/day)	76.5 (66.7)	74.2 (50.9)	74.9 (65.5)	69.6 (57.9)	−4.6 (−16.9–7.9)	0.465
Refined grains (g/day)	174 (88.4)	−118 (161)	176 (107)	−122 (171)	−4.0 (−41.7–33.7)	0.834
Nuts and seeds (g/day)	6.8 (8.0)	11.2 (7.8)	6.8 (9.7)	12.6 (8.2)	1.4 (−0.4–3.2)	0.130
Low fat dairy products (g/day)	275 (195)	53.9 (197)	256 (168)	57.9 (178)	4.0 (−38.7–46.7)	0.853
high fat dairy products (g/day)	229 (130)	−126 (63.7)	239 (126)	−135 (−63.4)	−9.0 (−23.4–5.4)	0.221
Fish (g/day)	5.5 (6.0)	3.5 (3.8)	5.8 (6.0)	3.2 (3.0)	−0.3 (−1.1–0.5)	0.448
sweets (g/day)	110.4 (68.4)	−72.4 (92.6)	100.1 (63.7)	−72.3 (76.7)	0.1 (−19.2–19.4)	0.991
Macro- and micronutrients intake						
Energy intake, kcal/day	2836 (600)	−522 (188)	2798 (536)	−554 (198)	−32.0 (−75.9–11.8)	0.152
Carbohydrate, % energy	58.9 (7.9)	−3.8 (6.2)	58.3 (7.3)	−3.9 (6.1)	−0.1 (−1.4–1.3)	0.888
Protein, % energy	10.0 (3.2)	4.3 (2.8)	11.1 (3.5)	3.9 (2.6)	−0.4 (−1.0–0.2)	0.200
Total fat, % energy	31.1 (6.7)	−2.3 (5.6)	30.6 (6.9)	−2.6 (5.7)	−0.3 (−1.6–1.0)	0.646
SFA, % energy	9.8 (2.2)	−2.1 (1.6)	9.4 (2.2)	−3.7 (1.8)	−1.6 (−1.9–−1.2)	< 0.001
MUFA, % of energy	16.0 (2.3)	3.7 (3.5)	16.4 (3.0)	3.8 (3.9)	0.1 (−0.7–0.9)	0.815
PUFA, % of energy	7.6 (1.7)	2.5 (3.5)	7.8 (2.0)	2.3 (3.5)	−0.2 (−0.9–0.6)	0.621
Fiber, g/day	33.4 (13.4)	3.5 (13.0)	30.6 (13.3)	8.6 (13.9)	5.1 (2.0–8.1)	0.001
cholesterol, g/day	248 (71.2)	−49.4 (70.6)	255 (72.5)	−70.3 (73.9)	−20.9 (−37.3–−4.4)	0.012
Sodium intake, mg/day	3048 (1826)	−658 (1946)	3214 (1960)	−713 (1801)	−55.0 (−481–371)	0.799

*DASH* dietary approach to stop hypertension, *CI* confidence interval, *SFA* saturated fatty acid, *MUFA* mono-unsaturated fatty acid, *PUFA* poly-unsaturated fatty acid

Data are mean (SD) unless otherwise indicated

Data adjusted for baseline values, energy intake and changes in physical activity level

P values were calculated by ANCOVA

At baseline, there were no significant differences in SBP, DBP, urine electrolytes among the two intervention groups. A reduction in SBP (mean change difference between diets −3.8, 95% CI −6.2 to −1.6 mmHg, *P* = 0.002) and urinary sodium (−2.3, 95% CI −3.7 to −0.9 mmol/dL, *P* = 0.001) was found in the legume-based DASH diet compared to the DASH diet group (Table 3).

**Table 3:** Baseline and the 16-week change in blood pressure and urinary sodium, urinary potassium and urinary sodium to potassium ratio after the DASH diet and legume-based DASH diet, based on intention to treat analysis

	DASH diet (n = 150)		Legume-based DASH diet (n = 150)		Difference in change	
	Baseline	Change from baseline	Baseline	Change from baseline	Mean (95% CI)	*P * value
SBP, mmHg	129.9 (15.2)	−6.1 (11.0)	130.9 (15.4)	−9.9 (10.5)	−3.8 (−6.2–−1.6)	0.002
DBP, mmHg	81.1 (9.0)	−2.3 (6.9)	81.6 (10.0)	−2.4 (8.0)	−0.1 (−1.8–1.6)	0.909
Urinary sodium (mmol/dL)	144.1 (28.9)	−4.1 (5.7)	141.9 (38.8)	−6.4 (6.5)	−2.3 (−3.7–−0.9)	0.001
Urinary potassium (mmol/dL)	52.1 (20.0)	20.3 (8.1)	51.5 (20.4)	20.5 (8.6)	0.2 (−1.7–2.1)	0.835
Urinary sodium to potassium ratio	3.0 (1.1)	−1.03 (0.6)	3.0 (1.1)	−1.05 (0.6)	−0.02 (−0.15–0.11)	0.773

*DASH* dietary approach to stop hypertension, *SBP* systolic blood pressure, *DBP* diastolic blood pressure

Data are express as mean (SD) unless otherwise indicated

Data adjusted for baseline values, oral anti-diabetic medications, antihypertensive drugs, and changes in physical activity level

P values were calculated by ANCOVA

**Fig. 2:**
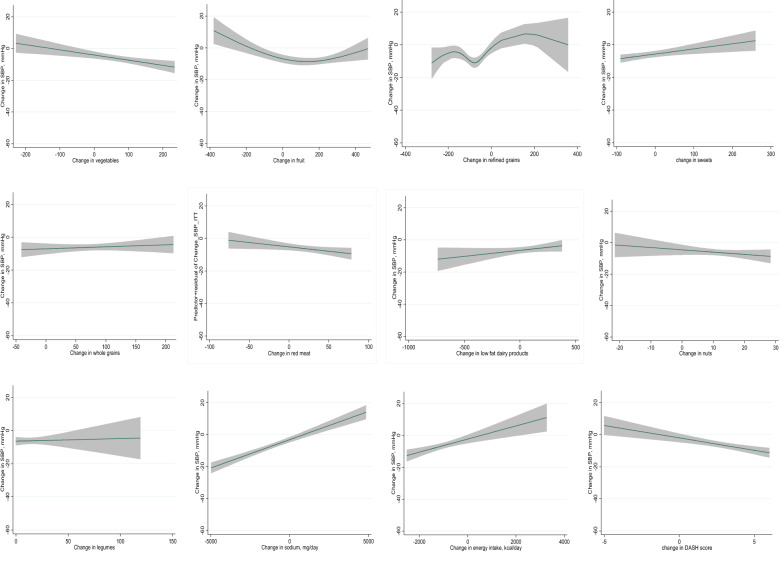
Change in systolic blood pressure according to change in food groups in the dietary approaches to stop hypertension diet group in overweight and obese type 2 diabetic patients

**Fig. 3:**
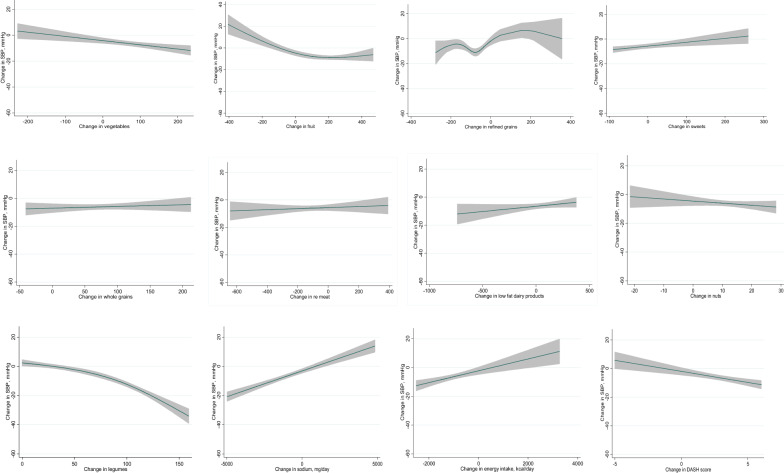
Change in systolic blood pressure according to change in food groups in the legume-based dietary approaches to stop hypertension diet group in overweight and obese type 2 diabetic patients

The multivariable RCS analysis showed the U-shaped association of refined grains intake (*P*-nonlinear < 0.001 in both intervention groups) with SBP. The greatest decrease in blood pressure was observed when reducing the intake of refined grains by −29.8 to −76.2 g/day in both intervention groups. Moreover, an L-shaped relationship was found between fruit intake and SBP in both intervention groups (*P* nonlinearity < 0.001 in both intervention groups). The SBP decreased with increasing fruit intake until 70.8 g/day in the DASH diet and 127.6 g/day in the legume-based DASH diet and then flattened. In the legume-based DASH diet, the SBP decreased with the legumes intake of more than 95.8 g/day (*P* nonlinear < 0.001). Vegetable and DASH score had a negative linear correlation with SBP. Sweet, energy, and sodium intake had a positive linear correlation with SBP in both intervention groups. No significant correlation was found between SBP and a change in the intake of whole grains, red meat, low-fat dairy products, and nuts in both intervention group (Figs. 2 and 3).

## Discussion

In the current trial among obese and overweight type 2 diabetic participants, the SBP was reduced by replacing one serving of red meat with legumes in the DASH diet at least 5 days a week over 16 weeks. Further investigation of food groups in the DASH diet showed that SBP decreased with increasing vegetable intake, while it increased with increasing intake of energy, sodium, and sweets. Moreover, the lowest SBP was observed when reducing the intake of refined grains by 29.8–76.2 g/day and increasing the intake of legumes by more than 98 g/day. Additionally, when the intake of fruits exceeded certain thresholds, blood pressure did not decrease.

In the current study, adopting a traditional DASH diet improved SBP among participants with type 2 diabetes. Our findings are in line with two meta-analyses indicating that DASH diet was associated with a −4.70 mmHg (−5.76 to −3.63 mmHg) change in SBP; however, despite −3.75 mmHg (−4.53 to −2.97 mmHg) change in DBP in the meta-analysis, no reduction in DBP was observed in the current study [25, 26]. Additionally, we found that replacing a serving of total red meat with legumes significantly reduced SBP compared to the traditional DASH diet. Contradicting our findings, adopting dietary patterns such as the Mediterranean and DASH diets, independent of red meat intake, improved blood pressure among healthy, overweight and obese adults [27], and hypertensive subjects [13]. In all of these studies, however, red meat was replaced with other animal protein sources. In the current study, when we further investigated which individual dietary food groups in the DASH diet contributed to blood pressure reduction, we found that changes in the red meat intake were not associated with SBP, but the legume intake more than 98 g decreased blood pressure. In a systematic review and meta-analysis of randomized controlled trials, there was no significant effect of substituting red meat with high-quality plant protein sources on blood pressure [16], which may be because the health-promoting effects of legume consumption, as seen in the current study, are achieved by consuming more than one serving per day [17].

In the current study, SBP decreased by lowering refined grains intake from ~ 30 (1 serving/day) to ~ 80 (2.5 servings/day) g/day. Previous systematic review and meta-analyses, and long-term follow-up studies have reported that increasing the consumption of refined grains and sweets from 1 to 3 servings per day had no association with hypertension [28–30]. This may be because the detrimental effects of consuming refined grains on chronic diseases, as seen in the current study, are observed when the intake of refined grains exceeds 200 g/day [31]. This is in line with our study in which sweets and refined grains were consumed more than 200 g/day. However, the U-shaped effect of reducing refined grains on SBP, both in the current study and a previous study [32], aligns with recommendations that emphasize the balanced consumption of whole grains and refined grains in the diet [33]. In addition, despite meta-analysis studies that reported the consumption of whole grains as 50–100 g/day reduces the risk of chronic disease such as hypertension, type 2 diabetes, and mortality [28, 31, 34], in the current study, increasing whole grain consumption did not affect blood pressure. Further studies are needed to be clarify the type and amount of grains for preventing and managing hypertension.

Our findings on the effect of increasing fruit and vegetable intake on lowering SBP are consistent with the meta-analysis of prospective cohort studies and randomized controlled trials, which concluded that fruits and vegetables were more effective in reducing SBP [25, 28, 35] and this relationship was linear for vegetables and nonlinear for fruits [28]. These findings are similar to our study, which showed that vegetable consumption was linearly related to SBP reduction, but the consumption of fruits to a certain threshold reduced SBP.

Previous clinical trials, investigating the effect of an energy restricted diet on blood pressure found that a low-energy diet reduces SBP and DBP [36, 37]. Consistently, our findings further confirm the inverse linear association between energy intake and SBP.

The strength of the current study was the interpretation of the results based on the ITT principles, low dropout rate, corrections for multiple tests, long-term nutritional intervention, and randomized trial design that allowed for controlling the confounders. Nevertheless, this study also has several limitations.

The participants were not blinded to the aim of the investigation (focused on increasing the intake of legumes), which may have affected their behaviors. Dietary interventions were recommended to participants, and no meals were prepared for them; therefore, the goal of the diet might not be fully attained. For this reason, the dietitian called the participants once a week and encouraged adherence to the dietary recommendations. We also gathered the food records of participants and assessed their adherence to dietary recommendations. However, these measures are insufficient for evaluating adherence to dietary interventions, and the assessment of the biochemical index of legume intake could be a more appropriate indicator. Also, financial constraints limited us to assessing dietary adherence accurately. Overall, adherence to a healthy diet is affected by socioeconomic status. This study was conducted in an area with a middle to high socioeconomic status, and our findings cannot be generalized to type 2 diabetic patients with a low socioeconomic status.

## Conclusions

The DASH diet, enriched in legumes, could improve SBP in participants with type 2 diabetes. In DASH diet, the balance of consumption of refined grains and fruits along with higher consumption of vegetables and lower consumption of sweets, sodium and energy reduced SBP.

## Data Availability

The datasets generated and/or analysed during the current study are not publicly available due institution’s policy but are available from the corresponding author on reasonable request.

## Abbreviations

CVD: Cardiovascular disease
DASH: Dietary approach to stop hypertension
RCT: Randomized controlled trial
IRCT: Iranian registry of clinical trials
TLGS: Tehran lipid and glucose study
ITT: Intention-to-treat
MICE: Multiple imputation by chained equations
ANCOVA: Analysis of covariance
SBP: Systolic blood pressure
DBP: Diastolic blood pressure
RCS: Restricted cubic spline
